# SHH Protein Variance in the Limb Bud Is Constrained by Feedback Regulation and Correlates with Altered Digit Patterning

**DOI:** 10.1534/g3.116.033019

**Published:** 2017-01-26

**Authors:** Rui Zhang, Chanmi Lee, Lisa Y. Lawson, Lillian J. Svete, Lauren M. McIntyre, Brian D. Harfe

**Affiliations:** * Department of Molecular Genetics & Microbiology, College of Medicine, University of Florida, Gainesville, Florida 32610; † Genetics Institute, University of Florida, Gainesville, Florida 32610

**Keywords:** Shh, limb, AER

## Abstract

mRNA variance has been proposed to play key roles in normal development, population fitness, adaptability, and disease. While variance in gene expression levels may be beneficial for certain cellular processes, for example in a cell’s ability to respond to external stimuli, variance may be detrimental for the development of some organs. In the bilaterally symmetric vertebrate limb buds, the amount of Sonic Hedgehog (SHH) protein present at specific stages of development is essential to ensure proper patterning of this structure. To our surprise, we found that SHH protein variance is present during the first 10 hr of limb development. The variance is virtually eliminated after the first 10 hr of limb development. By examining mutant animals, we determined that the ability of the limb bud apical ectodermal ridge (AER) to respond to SHH protein was required for reducing SHH variance during limb formation. One consequence of the failure to eliminate variance in SHH protein was the presence of polydactyly and an increase in digit length. These data suggest a potential novel mechanism in which alterations in SHH variance during evolution may have driven changes in limb patterning and digit length.

Historically, developmental biologists have viewed gene expression levels as being fixed within a given tissue at a specific time point. However, at the mRNA level, it is now clear that variance in mRNA expression occurs in numerous tissues (Levsky *et al.* 2002; Ozbudak *et al.* 2002; Mar *et al.* 2006, 2011). In this context, variance refers to cells that are perceived as being an identical age and type having a different amount of mRNA transcripts. This is the same as the standard statistical definition of variance. The variation of gene expression across populations, cell lines, and even within “identical” cells of a tissue can result in the production of substantially different phenotypes (Raser and O’Shea 2005; Raj *et al.* 2010). Variance in mRNA levels has also been found in genetically identical animals and may be one cause of reported differences in the penetrance of a given phenotype within inbred lines (Raser and O’Shea 2005). While a certain level of variance has been reported to be required in some pathways, variance in mRNA expression of core signaling pathways appears to be constrained (Mar *et al.* 2011).

Variance in mRNA levels could result in variance in the level of proteins produced in a tissue; however, this hypothesis has been difficult to test. Using the mouse and chick model systems, we have determined that variance in SHH protein level, a key signaling protein responsible for patterning a large number of tissues (McMahon *et al.* 2003), occurs within the limb bud at early stages of development. This is surprising since all current models of digit patterning propose that SHH protein levels are tightly linked to digit identity (Bastida and Ros 2008; Zeller *et al.* 2009).

In this report, we found that variance in SHH levels was reduced ∼10 hr after the limb bud formed, suggesting that constrained SHH protein levels may be essential for normal limb outgrowth. By eliminating the ability of a region of the limb bud ectoderm called the AER to respond to SHH protein levels, SHH variance was unconstrained. In these animals, digit length increased suggesting the possibility that a specific target amount of SHH protein is required for normal limb development.

## Materials and Methods

### Mice

The *Shhgfpcre*, *Msx2-Cre*, and *Smo^flox^* alleles have been described previously (Sun *et al.* 2000; Zhang *et al.* 2001; Harfe *et al.* 2004; Nolan-Stevaux *et al.* 2009) and were maintained on mixed genetic backgrounds. Genotyping was performed with DNA extracted from tail or yolk sack tissue. Embryos of the genotype *Smo^flox/flox^* or *Smo^flox/+^*were phenotypically indistinguishable from normal mice and used as controls along with wild-type embryos. Animals were handled according to the guidelines of the University of Florida Institutional Animal Care and Use Committee (protocol number 201005047).

### Whole-mount RNA in situ hybridization

RNA *in situ* hybridization was performed as previously described (Bouldin *et al.* 2010). At least three embryos of the same genotype or somite stage were examined in all experiments.

### quantitative real-time PCR (qRT-PCR) analysis

Contralateral forelimb buds from 18 different 32ss mouse embryos were dissected and lysed separately in RLT Plus buffer (QIAGEN, Germantown, MD) supplemented with 4 ng/μl of β-mercaptoethanol. Total RNA was isolated from individual limb buds using a RNeasy Plus Micro kit (QIAGEN) and reverse transcribed into cDNA using a SuperScript First-Strand Synthesis System for RT-PCR (Invitrogen, Carlsbad, CA), following the manufacturer’s instructions. qRT-PCR was performed on a CFX96 Real-Time System + C1000 Thermal Cycler (Bio-Rad, Hercules, CA) with iQ SYBR Green Supermix (Bio-Rad) using a two-step amplification (95° for 15 sec, 60° for 1 min, 40 PCR cycles). Each cDNA sample was run in triplicate. Primer sequences used were: Shh-F, 5ʹ-5ʹ-CCGAACGATTTAAGGAACTCACCC-3ʹ; Shh-R, TGGTTCATCACAGAGATGGCCAAG-3ʹ; Gapdh-F, 5ʹ-CCAAGGTCATCCATGACAACT-3ʹ; and Gapdh-R, 5ʹ-ATCACGCCACAGCTTTCC-3ʹ.

### Tissue preparation and western blotting

Embryos were staged by counting somites. Limb buds were harvested by dissecting them from the body trunk. Individual limb buds were lysed in 10 μl of M-PER mammalian protein extraction reagent (Thermo Scientific, Rockford, IL) supplemented with 1 × Halt protease inhibitor cocktail and 5 mM EDTA (Thermo Scientific) and stored at −20°. When analyzed, each sample was supplemented with an additional 10 μl of Laemmli sample buffer (Bio-Rad), boiled for 10 min at 95°, resolved on 12.5% SDS-PAGE, and transferred to a PVDF membrane. Contralateral limb buds were loaded side-by-side on the same gel to eliminate variations in experimental conditions. Immunoblotting was performed as previously described (Schiapparelli *et al.* 2011) using anti-SHH (1:2000 dilution, sc-9024, Santa Cruz) and anti-glyceraldehyde 3-phosphate dehydrogenase (GAPDH) (1:20,000 dilution, ab8245, Abcam) antibodies, and detected with peroxidase-conjugated goat anti-rabbit IgG (Jackson ImmunoResearch, West Grove, PA) and sheep anti-mouse IgG (GE healthcare, Pittsburgh, PA) secondary antibodies.

### Imaging and quantification of SHH and GAPDH blots

The SHH and GAPDH immuno-bands were visualized with Western Lightning Ultra chemiluminescence substrate (Perkin Elmer, Waltham, MA) and detected using a ChemiDoc XRS imager (Bio-Rad). Quantification was performed using Quantity One 1-D analysis software (Bio-Rad). Signal intensities (I) of each band were determined using volume analysis with local object background correction applied. The variances (V) of SHH or GAPDH levels between pairs of limbs (right/left) were calculated as the variance for the ratio of right/left for SHH and GAPDH separately. For these experiments, five to nine embryos were collected per somite stage, and for each embryo total protein levels for SHH and GAPDH were measured in all four limbs.

### SHH protein dilutions

*Shh*-null embryos were generated by intercrossing *Shhgfpcre/+* mice and harvested at embryonic day (E)10–10.5. To prepare SHH-containing or SHH-lacking limb extracts, 24 fore- and hindlimb buds from phenotypically normal or *Shh*-null embryos were collected, respectively, and pooled and lyzed as described above. For dilutions, between 2 and 25 μl of SHH-containing lysates were mixed with 5 μl of SHH-lacking lysate. Conversely, 15 μl of SHH-containing lysate was mixed with increasing amounts of SHH-lacking lysates ranging from 2 to 15 μl. PBS was used to ensure that each lane was loaded with 30 μl. Each dilution was examined on western blots in triplicates To test the validity of quantification on western blots, 1 mg of SHH lyophilized powder was resuspended in BSA supplemented with 1 × Halt protease inhibitor cocktail and 5 mM EDTA (Thermo Scientific). Serial dilutions were loaded on 12.5% SDS-PAGE gels in varying concentrations (150, 250, 350, and 450 pg). Western blotting and quantification were performed as described above. The experiments were repeated six times and the measurement error was calculated as described below.

### Statistics

An *F* test for homogeneity of variance was used to test the null hypothesis that the variance between SHH and GAPDH were equal. To estimate the variance, a mixed model was used where the ratio of right to left was the dependent variable and the gene was the independent variable. We fitted a block diagonal matrix where each gene and stage was specified separately for the variance/covariance matrix and used REML to estimate the variance components (McCullagh and Nelder 1989). Comparisons of the variance in the right/left ratio for proteins between the wild-type and *Msx2-Cre*; *Smo^flox/flox^* or S*hhgfpcre/+* mutants were conducted using the folded *F* in a simple model, where only the fixed effect of a genotype was considered. In order to test the null hypothesis that there was no difference in the amount of protein between the right and the left limb buds, a sign test was used. Individual embryos were scored as left biased if the left side had more SHH relative the right after adjusting for GAPDH. Under a random model, we expect that half of the embryos should be left biased and half right biased. To test whether right/left bias was different from a frequency of 0.5, the sign test was performed using the null hypothesis of *P* = 0.5.

### Ethics statement

No human subjects were used in the experiments described in this manuscript. Mice were handled according to the guidelines of the University of Florida Institutional Animal Care and Use Committee (protocol number 201005047). Euthanasia was performed by cervical dislocation as described in our animal protocol.

### Data availability

**Figure 1:**
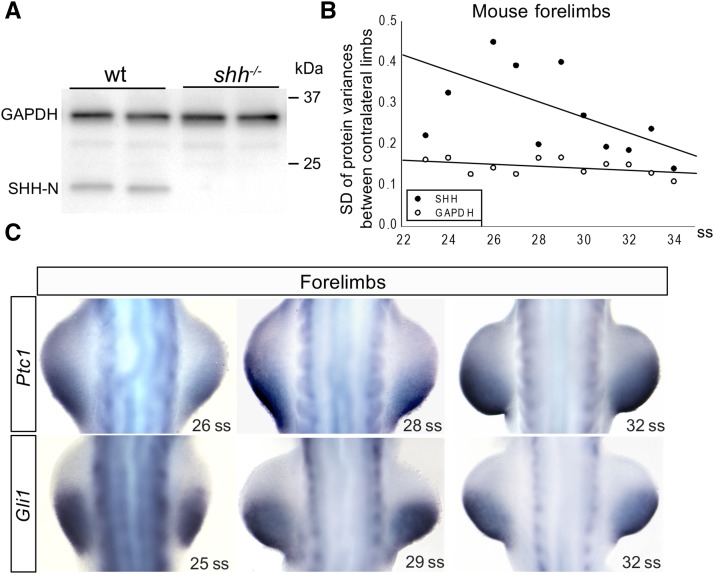
Variance in SHH protein levels is reduced by 27ss. (A) Western blots for SHH and GAPDH in wild-type and *Shh^−/−^* mouse limb buds. The absence of a ∼19 kDa band in the *Shh^−/−^* mouse limb buds demonstrates the specificity of the α-SHH antibody. (B) Comparisons of variance in SHH and GAPDH protein levels between contralateral forelimbs from the same mouse embryo at stage 23ss–34ss. The earliest time point at which SHH protein was detected on western blots was 23ss (∼E9.25). Each point represents the SD of the left *vs.* right (R/L) ratio from at least five embryos at the same ss. The data were fitted with a linear regression model. (C) *Ptc1* and *Gli1* expression in mouse forelimbs at different embryonic stages. At 26ss, clear differences in *Ptc1* and *Gli1* mRNA levels were observed. These differences decreased at later somite stages. E, embryonic day; GAPDH, glyceraldehyde 3-phosphate dehydrogenase; mRNA, messenger RNA; SHH, Sonic Hedgehog; ss, somite stage; wt, wild-type.

Supplemental data included in this proposal (Supplemental Material, Figure S1, Figure S2, Figure S3, and Figure S4) provide additional evidence that our measurements of SHH proteins levels are quantitative. In addition, measurements of protein levels in limb buds of chickens are shown. These data complement the mouse data shown in Figure 1. 

## Results and Discussion

In the limb bud, *Shh* is produced by cells in the distal posterior region of the limb bud called the Zone of Polarizing Activity (ZPA) (Riddle *et al.* 1993). Previous work demonstrated that a limb bud-specific enhancer called the ZPA enhancer element (ZRS) is required and sufficient to transcribe *Shh* mRNA in the limb bud ZPA (Lettice *et al.* 2003, 2008; Sagai *et al.* 2005). In addition, alterations in the amount of SHH protein present in the limb bud cause defects in limb patterning and growth (Tickle 1981; Riddle *et al.* 1993; Yang *et al.* 1997; Sanz-Ezquerro and Tickle 2000).

In mice, *Shh* expression is first detected in the posterior forelimb bud at ∼E9.5 and in the hindlimb bud at ∼E10.0 (Bueno *et al.* 1996; Buscher *et al.* 1997; Lewis *et al.* 2001; Zhu *et al.* 2008). Previous experiments have demonstrated that tight regulation of SHH protein in a limb bud is essential for normal pattern formation (Tickle *et al.* 1975; Tickle 1981; Riddle *et al.* 1993; Chang *et al.* 1994; Yang *et al.* 1997). However, it is unknown how SHH protein levels are initially specified and how the concentration of SHH protein required for normal patterning is maintained during limb bud growth. We investigated SHH protein levels in normal development by measuring SHH levels in individual mouse and chick limb buds in both fore- and hindlimbs using western blots during somite stages (ss) 22–40 (approximately mouse E9.25–11.0).

The ideal way to examine differences in protein amounts is to use tissue from the same animal since all tissues within an animal are, with a few exceptions, genetically identical and at the same age. In our experiments, SHH protein levels were compared between limb buds within the same embryo, thus eliminating potential issues regarding the age of the two samples being compared.

If SHH protein levels were identical in the left and right fore- or hindlimbs within an embryo at all stages of development, this would suggest that SHH levels do not deviate bilaterally during limb development. In contrast, if SHH protein levels were found to be different between two limb buds of the same animal, these data would suggest that bilateral deviations in SHH protein levels occurred. Further, the bilateral deviation may play an important role in limb patterning.

It is important to note that only a “snap-shot” of the level of SHH protein at any given somite stage can be determined within an embryo. For example, if bilateral deviations occurred by chance, in some embryos the amount of SHH protein in the left and right limb buds would be identical at the time point the embryo was analyzed. By examining multiple embryos, deviations in bilateral SHH protein levels can be determined. In all experiments, SHH protein levels were compared between the two forelimbs or hindlimbs within a given embryo since, in both the mouse and chick model systems, fore- and hindlimbs develop at different rates (Martin 1990; Hamburger and Hamilton 1992).

Levels of SHH in individual limb buds were quantified using an antibody specific for the 19-kDa processed form of SHH (Figure 1A). The 19-kDa form of SHH has been shown to be responsible for activating the hedgehog (Hh) signaling pathway (Goetz *et al.* 2002). To determine if the antibody was specific for SHH, western blots containing *Shh*-null limb buds were analyzed. In these mutant limbs, loss of SHH was observed (Figure 1A).

To validate that western blots were sensitive enough to detect quantitative differences in protein levels of individual limb buds, a linear serial dilution analysis of SHH or GAPDH protein was performed. The serial dilutions using pure SHH protein were quantified by western blot and, using the known concentration as the dependent variable, a simple linear regression model was fitted. In these experiments, which were performed four times at each concentration, the concentration of protein loaded was a strong predictor of the amount of signal detected on the gel [Figure S1; $r_{\mathrm{GAPDH}}^{2}$ = 0.987 (*P* < 0.001) and $r_{\mathrm{Shh}}^{2}$ = 0.954 (*P* < 0.001)]. The western blot band intensities of SHH protein quantified from an individual limb bud was equivalent to the amount of SHH present in the 10–20 μl serial dilution analysis (this depended on the age of the limb bud). These data indicate that SHH protein levels can be reproducibly measured from individual limb buds.

To determine if the level of SHH protein between limb buds of the same embryo is variable, individual limb buds from CD1 mouse fore- and hindlimbs were collected. Limb buds from five to nine individual embryos were collected at each somite stage (23–39ss). Dissections by even the most skilled scientists can potentially be unintentionally biased by the method that is used to collect samples. In our report, all mouse dissections were done by one scientist and all chick dissections by a second scientist (see below and Figure S2). Both scientists were right handed.

To test for bias, which was one of our first quality control tests, we tested the null hypothesis that SHH protein levels were symmetrical. In our experiments, there was no consistent bias for elevated (or reduced) amounts of SHH protein to the left or right forelimb (*n* = 87, *P* = 0.098, sign test) or hindlimb (*n* = 84, *P* = 0.543). The random distribution of elevated SHH protein levels in either the right or left limbs was found in both chick and mouse embryos. Therefore, we concluded that the method used to dissect limb buds did not bias the amount of SHH protein detected in left *vs.* right limb buds.

To determine if our dissections included all limb bud *Shh*-expression, and no *Shh*-expressing tissue from the embryo flank, we examined *Shh* mRNA expression *in viv*o. In these experiments, limb buds from wild-type 27ss or 33ss mouse embryos were dissected from the flank of the embryo and then analyzed for *Shh* expression using RNA *in situ* hybridization (Figure S4). These dissections, as well as the dissections used to obtain tissue for the individual limb bud western blots, were performed in an identical manner. These experiments demonstrated that the entire limb domain of *Shh* expression was captured in the dissected limb buds. *Shh* expression from the floor plate, notochord, and/or gut was not captured in our dissections (Figure S4). Previously, we reported that at E10.5, all SHH protein is in the posterior distal region of the limb bud [Bouldin *et al.* 2010] and therefore would be fully captured in our dissections. We and others have not been able to detect SHH protein on tissue sections of pre-E10.5 limb buds, although our western blot analysis clearly demonstrates that SHH protein is present in the early limb bud.

The difference in SHH protein levels between contralateral limb buds of the same embryo was quantified (see *Materials and Methods*). GAPDH was used as a control (Figure 1B). SHH had a significantly different variance of the right/left ratio compared to GAPDH for both fore- (*P* < 0.001) and hindlimbs (*P* < 0.001). Examination of the SD for each somite stage showed a downward trend in SHH that was not apparent in GAPDH (Figure 1B and Figure S2A).

At all stages, GAPDH was used to determine the amount of tissue that was dissected. The low amount of variance in GAPDH levels suggests that our dissections captured essentially equivalent amounts of tissue from each limb bud of a given embryo (each lane of the western blots included a single left or right limb bud and no pooling of samples occurred). It is important to note that no normalization of protein was performed in our experiments. The amount of SHH protein measured in each limb bud was the total amount present in the limb bud for a given stage. These data support our view that equivalent amounts of limb bud tissue were present in each sample analyzed.

A comparison of protein levels in “early” (23–26ss embryos) with “late” (> 27ss embryos) was performed to determine if variance changed as development progressed. Variance in the right/left ratio for GAPDH in the forelimb was not significantly different between early (*n* = 34) and late (*n* = 53) somite stages (*P* = 0.700; ${\hat{\sigma }}_{late}^{2}$ = 0.025 compared to ${\hat{\sigma }}_{early}^{2}$ = 0.023). However, variance of the Right/Left ratio for SHH protein levels was found to differ between early and late stages (*P* = 0.006; ${\hat{\sigma }}_{late}^{2}$ = 0.06 compared to ${\hat{\sigma }}_{early}^{2}$ = 0.15). This indicates that the amount of SHH protein in the same embryo can deviate between the left and right limb buds during the period of early development, which is ∼10 hr after limb formation commenced.

During early embryonic development, a number of genes are transcribed in an invariant asymmetrical pattern on either the right or left side of the embryo (Lee and Anderson 2008). The asymmetrical expression of genes in the early embryo is essential for normal patterning of internal organs. In the limb bud, SHH protein levels were not found to be consistently asymmetrical to the left or right side in either fore- (*n* = 87, *P* = 0.098, sign test, see *Materials and Methods*) or hindlimbs (*n* = 84, *P* = 0.543). These data suggest that variance in the ratio of right/left SHH protein levels is not connected to the molecular pathways that establish right/left asymmetry during early embryonic development.

To further investigate the differences in SHH protein levels uncovered in the western blots, *Gli1* and *Ptch1* mRNA expression were examined *in vivo*. These genes are direct transcriptional targets of the Hh signal transduction pathway and have been reported to serve as sensitive readouts of SHH signaling activity (Marigo *et al.* 1996; Marigo and Tabin 1996; Lee *et al.* 1997; Pearse *et al.* 2001). RNA *in situ* hybridization using *Gli1* or *Ptch1* riboprobes showed visible differences in mRNA transcription domains of these two genes in right and left limb buds of the early (23ss–27ss) embryos. These transcription differences decreased between 27ss and 31ss and no visible differences in mRNA expression domains of these genes were detected late (>27ss; Figure 1C). These results support a model in which different amounts of Hh signaling occur in the right and left limb buds of the same wild-type embryo prior to 27ss. After 27ss, SHH protein levels and Hh signaling are present at the same level in both forelimbs. Identical results were obtained in mouse hindlimbs (Figure S2A) and using the chick model system (Figure S2, B–D). These data suggest that a molecular mechanism is present in the limb bud that is responsible for quickly detecting and modulating levels of SHH as development progresses.

We recently reported that SHH protein present in the AER activates the Hh signaling pathway in this tissue (Bouldin *et al.* 2010). Removal of the ability of the AER to activate Hh signaling resulted in an expanded domain of *Shh* mRNA transcription in the ZPA region of the limb bud and elevated expression of *Fgf*s in the AER (Bouldin *et al.* 2010). The reported phenotypic consequence of loss of Hh signaling in the AER was postaxial polydactyl (Bouldin *et al.* 2010).

**Figure 2:**
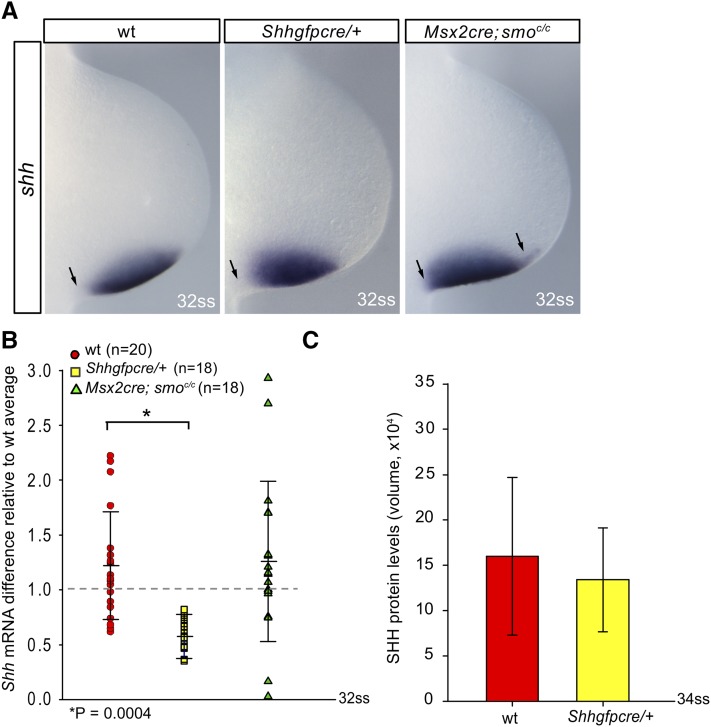
Analysis and quantification of *Shh* mRNA levels. (A–C) *Shh* RNA *in situ* hybridization in control (A), *Shhgfpcre/+* (B), and *Msx2-Cre*; *Smo^flox/flox^* limb buds (C), showing proximally expanded *Shh* expression in *Msx2-Cre*; *Smo^flox/flox^* forelimbs. (D) qPCR of *Shh* mRNA levels in mutant and control forelimb buds. Each data point represents the mRNA fold difference of individual limb buds compared with the average *Shh* mRNA level from 20 wt limb buds. A dashed line denotes the average *Shh* mRNA level. Limb buds that contained more than the average were above the line and those that contained less *Shh* mRNA than the average were below the dotted line. (E) Comparisons of SHH protein levels in *Shhgfpcre/+* (*n* = 27) and control forelimb buds (*n* = 24). The relative SHH amounts were measured by measuring the intensity of bands on western blots (see *Materials and Methods*). The data were collected from eight replicates. Error bars represent two SDs from the mean. mRNA, messenger RNA; qPCR, quantitative polymerase chain reaction; SHH, Sonic Hedgehog; ss, somite stage; wt, wild-type.

To determine if the ability of the AER to sense SHH protein levels was required for reduction of the deviation of SHH protein between limb buds at later somite stages, we conditionally deleted a floxed allele of *Smoothened* (*Smo*) in the AER using the *Msx2-Cre* allele that we previously reported (Bouldin *et al.* 2010). Removal of *Smo* (*Msx2-Cre*; *Smo^flox/flox^* embryos) resulted in loss of Hh signaling in the AER, and proximal and distal expansion of *Shh* mRNA expression in the limb bud mesenchyme (Figure 2, A–C and Bouldin *et al.* 2010). Embryos null for a single allele of *Shh* expressed *Shh* in an expression pattern similar to wild-type embryos (Figure 2B). In particular, neither proximal nor distal expansion of *Shh* mRNA was observed. The subtle differences in animals heterozygous for *Shh* could be due to slight differences in ages between the two limb buds. Heterozygous *Shh*-null mice are phenotypically wild-type in all tissues that have been examined to date, including the limb (Chiang *et al.* 1996, 2001; Harfe *et al.* 2004).

To determine if the observed expanded domain of *Shh* mRNA in limb buds in which Hh signaling was removed from the AER resulted in an increase in the amount of *Shh* mRNA produced from the *Shh* locus, qRT-PCR analysis was performed. Surprisingly, the amount of *Shh* mRNA produced in *Msx2-Cre*; *Smo^flox/flox^* limbs (*n* = 18) was not significantly different (*P* = 0.796) to that in control forelimbs (*n* = 20), even though RNA *in situ* hybridization analysis revealed a larger domain of *Shh* mRNA expression (Figure 2D). These data indicate that the broader domain of *Shh* expression observed upon removal of the Hh signaling pathway in the AER does not result in a significant increase in the total number of *Shh* mRNA transcripts.

Removal of one functional allele of *Shh* does not result in the production of a visible phenotype (Chiang *et al.* 1996, 2001; Harfe *et al.* 2004). However, *Shh* mRNA expression in limb buds that contained only a single functional *Shh* allele (*Shhgfpcre/+* embryos) was found to yield a ∼50% reduction in *Shh* mRNA compared to control limb buds (*P* = 0.0004) (Figure 2D). In contrast, SHH protein production in heterozygous *Shhgfpce/+* forelimbs (*n* = 27) was not significantly different to the amount of SHH produced in control limbs (*n* = 24) (*P* = 0.360) (Figure 2E). The finding that SHH protein in *Shhgfpce/+* limbs retains a level comparable to that of the control limbs, even in the presence of a ∼50% reduction in *Shh* mRNA, suggests that *Shh* mRNA translation may play a key role in regulating the amount of Hh signaling produced in the vertebrate limb bud. These data are consistent with the absence of a phenotype in animals null for one allele of *Shh*.

**Figure 3:**
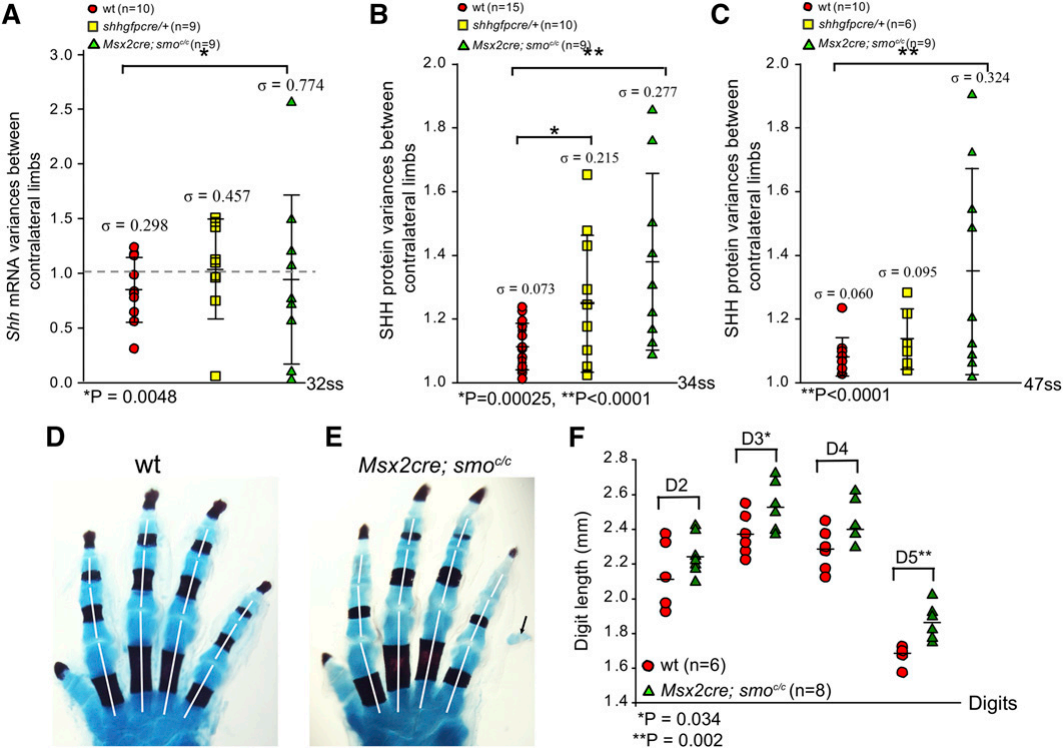
Comparisons of *Shh* mRNA levels or SHH protein levels between contralateral limb buds at different somite stages. (A) Variance of *Shh* mRNA between wt *Shhgfpcre/+* and *Msx2-Cre*; *Smo^flox/flox^* limb buds at 32ss. (B and C) SHH protein variances between limb buds at 34ss (B) and 47ss (C) in wt, *Shhgfpcre/+*, and *Msx2-Cre*; *Smo^flox/flox^* mutants. In (A)–(C), each data point represents the fold difference between paired limbs from the same embryo. A value of 1.0 corresponds to the same amount of *Shh* mRNA (A) or SHH protein (B and C) being present in both limb buds within the same embryo. Error bars represent two SDs from the mean. Skeletal preparation of control (D) and *Msx2-Cre*; *Smo^flox/flox^* (E) forelimbs. Measurement of the metacarpals and phalanges of digits 2–5 in newborn (P0) mouse forelimbs was performed. White lines depict the region of each digit that was measured, with the total length of each digit being the sum of the three segments. (F) Digits 2–5 (D2–D5) from six wt and eight mutant *Msx2-Cre*; *Smo^flox/flox^* embryos were measured. D3 (*P* = 0.034) and D5 (*P* = 0.002) were significantly longer in the mutants compared to controls. mRNA, messenger RNA; SHH, Sonic Hedgehog; ss, somite stage; wt, wild-type.

To determine if removal of one allele of *Shh* or the ability to respond to SHH ligand in the AER played a role in the elimination of variance in the right/left ratio, the ratio of *Shh* mRNA or SHH protein between limb buds of the same embryo was quantified. If removal of a single allele of *Shh* or removal of Hh signaling in the AER affected the ability of the limb bud to remove SHH protein variance, *Shh* mRNA and/or SHH protein levels would be expected to continue to vary in later stages. Indeed, the variance of the *Shh* mRNA right/left ratio of *Msx2-Cre*; *Smo^flox/flox^* (*n* = 9, ${\hat{\sigma }}^{2}$ = 0.60) was different from control embryos at late somite stages (*n* = 10, ${\hat{\sigma }}^{2}$ = 0.089, *P* = 0.0048; Figure 3A). Surprisingly, the variance in the right/left ratio for *Shhgfpcre/+* limb buds (*n* = 9, ${\hat{\sigma }}^{2}$ = 0.21) was not significantly different (*P* = 0.11) from the wild type at late somite stages (these limb buds contain approximately half the amount of RNA as controls, see Figure 2B). The observed estimates of the variance were consistent with additive effects of the two alleles, where the heterozygote had an estimated value halfway between the homozygotes.

In our analyses of SHH protein levels in mutant embryos, 34ss was analyzed instead of 32ss (the stage SHH protein levels are fixed in wild-type animals) to take into account possible delays in protein syntheses and post-translational modifications resulting from alterations in mRNA levels and/or the Hh signaling pathway in mutant backgrounds. Limb buds that lacked Hh signaling in the AER (*Msx2-Cre*; *Smo^flox/flox^*, *n* = 9, ${\hat{\sigma }}^{2}$ = 0.077) and limb buds that contained a single functional allele of *Shh* (*Shhgfpcre/+*, *n* = 10, ${\hat{\sigma }}^{2}$ = 0.046) each had a significantly greater variance in the right/left ratio of limb bud SHH protein levels within the same embryo at 34ss (*P* < 0.0001 and *P* = 0.00025, respectively) compared with wild-type control limbs (*n* = 15, ${\hat{\sigma }}^{2}$ = 0.005, Figure 3B). The variance in the right/left ratio for GAPDH was similar among all three genotypes (${\hat{\sigma }}^{2}$ = 0.014 for *Msx2-Cre*; *Smo^flox/flox^*, ${\hat{\sigma }}^{2}$ = 0.03 for *Shhgfpcre/+* and ${\hat{\sigma }}^{2}$ = 0.03 for control limbs).

The inability of *Shhgfpcre/+* and *Msx2-Cre*; *Smo^flox/flox^* limb buds to correctly specify normal levels of SHH protein, as indicated by variance in the right/left ratio of SHH protein, suggests that the presence of both *Shh* alleles and the ability of the AER to respond to SHH ligand is required for the reduction of SHH variance in the limb bud mesoderm.

Fluctuations in the right/left ratio of SHH protein levels occurred in mice that lacked a single allele of SHH at late stages (34ss); however, a limb phenotype was not observed in these mice. To determine if SHH protein levels in this genotype were set at correct levels at very late time points, SHH protein was measured in right and left limb buds of the same embryo at 47ss. At this stage, the variance of the right/left ratio in control limbs was (*n* = 10, ${\hat{\sigma }}^{2}$ = 0.0036), indicating that the variance in the right/left ratio of SHH protein levels was compatible with normal limb patterning. The variance of the right/left ratio in embryos lacking one allele of *Shh* was slightly higher at ${\hat{\sigma }}^{2}$ = 0.009 (*n* = 6), a level comparable to control limbs at 34ss (*P* = 0.11), whereas the variance in the right/left ratio for embryos lacking Hh signaling in the AER was significantly higher (*n* = 9, ${\hat{\sigma }}^{2}$= 0.105, *P* < 0.0001). These data suggest that variance in the right/left ratio of SHH protein levels, at least until 34ss, is not detrimental to limb patterning but that variance at late stages correlates with limb patterning defects.

To determine the consequence of not eliminating right/left differences in SHH protein, skeletons of *Msx2-Cre*; *Smo^flox/flox^* mutants, in which variance in right/left SHH protein ratio was abnormally maintained until at least 47ss (Figure 3C), were analyzed. In these animals, digits 3 and 5 were found to be significantly longer in mutant limbs compared to control limbs (Figure 3, D–F). Interestingly, these are the two SHH-dependent digits that form last during normal mouse limb development (Zhu *et al.* 2008), suggesting that variance in the right/left ratio of SHH could potentially affect digit length.

To ensure that our results were not a result of measurement error, we took several approaches. First, we considered whether the relationship between the variance in the right/left ratio and the somite stage was potentially an artifact of a mean/variance relationship. We find no relationship between the mean and SD for the right/left ratio (*ρ* = 0.08, *P* = 0.58).

Next, we performed a series of control experiments designed to quantify the measurement error in our methods. We determined the amount of SHH protein present in samples where the amount of SHH protein was fixed but the total amount of protein present was increased (in these experiments, protein extracted from *Shh*-null embryos were mixed with wild-type limb extract). Increasing non-SHH proteins did not change the amount of SHH protein detected in our experiments (Figure S1C, red line). In a separate experiment, we increased the amount of SHH-containing limb extract while maintaining a constant amount of SHH-lacking extract (protein extracted from *Shh*-null embryos was used). In this experiment, a linear increase in SHH protein was detected, demonstrating that western blots can be used to quantify increases in SHH protein levels (Figure S1C, black line). Finally, to estimate the measurement error directly on quantitative estimates of SHH protein, we analyzed increasing concentrations of pure SHH protein using western bots (Figure S1D). Known concentrations of pure SHH protein in varying amounts (150, 250, 350, and 450 pg) were loaded on six different blots and then measured using our protocol. These data show a clear linear relationship between the average amount of protein loaded and estimated signal intensity (*r*^2^ = 0.986, *P* = 0.005). For the six measures, we calculated the mean and variance at each known concentration. We compared all pairwise estimates using an *F* test for equality of variance. The variance at 250, 350, and 450 pg were not statistically different from each other. The variance at 150 pg SHH was significantly lower than the variance at the higher levels (250, 350, and 450 pg). The amount of SHH protein increases over somite stages, and if the measurement error was responsible for our observations, we would expect to see a higher variance at later somite stages, the opposite of what we observe. These experiments show that the measurement error is small and that SHH levels are detected reproducibly and quantitatively. Our observation that the variance in SHH between limb buds from the same embryo at earlier somite stages is higher is unlikely to be due to measurement error.

### Conclusions

SHH acts as a morphogen to direct autopod patterning and outgrowth [reviewed in Towers *et al.* (2012)]. Previous work has shown that the concentration of SHH protein is essential for normal limb patterning and outgrowth to occur. In this report, we show that in wild-type limb buds, SHH protein initially fluctuates between right and left, as demonstrated by an increase in protein variance of the right/left ratio, but that variance is drastically decreased ∼10 hr after the commencement of limb formation. Our analysis of embryos that are incapable of activating the Hh signaling pathway in the AER in response to SHH ligand reveals that Hh signaling in the AER is essential for the normal decrease of SHH protein variance in the limb bud.

Recently, a “two phase” model for initiating and then regulating the amount of *Shh* mRNA produced in the limb bud was proposed (Bénazet *et al.* 2009). In this model, a “fast” feedback loop occurs during the first 2 hr of limb development. During this time, GREM1 protein quickly downregulates BMP4 activity resulting in the initiation of *Shh* mRNA transcription in the ZPA. A “slow” *Shh*/*Grem1*/*Fgf* feedback loop is proposed to maintain *Shh* mRNA expression in the ZPA ∼12 hr later.

**Figure 4:**
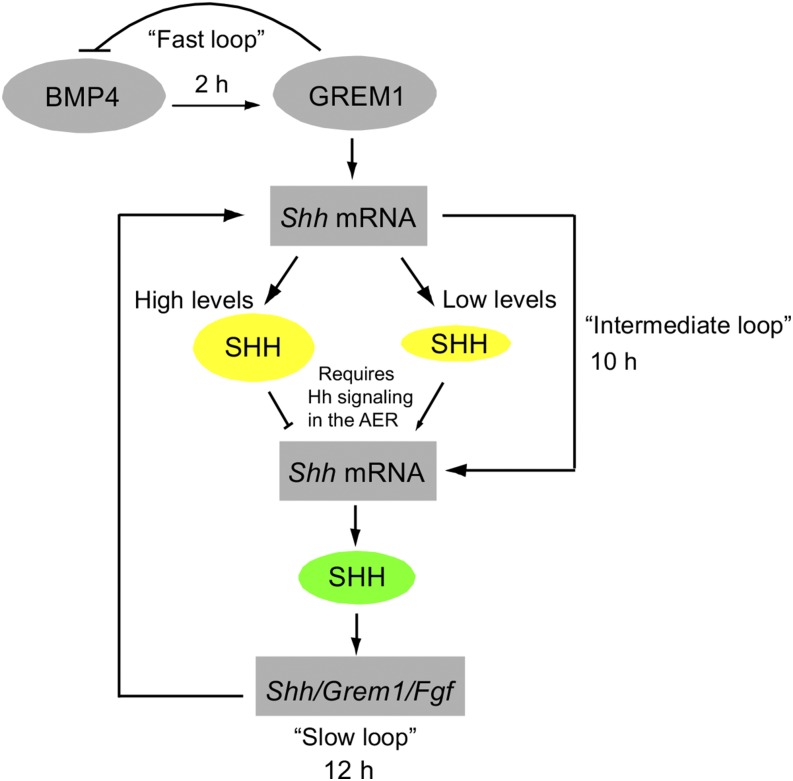
Regulation of SHH protein levels in the vertebrate limb bud. A “fast” feedback loop in which BMP4 activity is downregulated by GREM1 initiates transcription of *Shh* mRNA in the limb. The initiation of *Shh* mRNA transcription and/or translation of *Shh* mRNA into protein is insufficient to regulate SHH protein levels that are required for normal patterning. An “intermediate” SHH protein-dependent pathway functioning in the AER is required to specify SHH protein levels by ∼10 hr after limb bud initiation. ∼2 hr later, a “slow” *Shh/Grem1/Fgf* feedback loop is responsible for maintaining *Shh* mRNA transcription in the ZPA. The slow and intermediate pathways may continue to work during later development to ensure levels of SHH protein are produced that are compatible with normal development. The role SHH protein plays in the model is denoted by yellow and green circles. AER, apical ectodermal ridge; mRNA, messenger RNA; SHH, Sonic Hedgehog; ZPA, zone of polarizing activity.

Based on the data in our report, we propose that, after the fast BMP4/GREM1 feedback loop initiates transcription of *Shh* mRNA, an “intermediate” SHH protein-based feedback loop specifies the amount of SHH protein within the limb bud (Figure 4). In this model, the AER may respond to different amounts of SHH protein, which in turn could regulate transcription of *Shh* mRNA from the ZPA.

It is currently unknown how or what pathway(s) downstream of AER Hh signaling may function to regulate the amount of SHH protein produced in the limb bud mesenchyme. FGFs produced by the AER have been postulated to regulate transcription of *Shh* in the ZPA (Niswander *et al.* 1994); however, the molecular mechanism functioning downstream of AER-expressed FGFs is currently unknown. Our model suggests that the presence of an intermediate SHH protein feedback loop allows the limb bud to quickly specify the level of SHH protein required for normal outgrowth.

We attempted to measure FGF8 and FGF4 on the same blots in which SHH protein was quantified (levels of any protein analyzed would have to be performed on the same blot in which SHH and GAPDH protein was measured to enable comparisons to be made between limb buds of the same embryo). FGF4 is a target of SHH and would be expected to change as SHH changed. FGF8 has not been reported to be a direct SHH target. Unfortunately, we were not able to detect either FGF4 or FGF8 on our SHH/GAPDH western blots using numerous different antibodies.

The decrease in SHH variance for the right/left ratio observed over the first 10 hr of limb development places this feedback loop directly after *Shh* mRNA is initially transcribed and prior to initiation of the “slow” *Shh*/*Grem1*/*Fgf* feedback loop required to maintain *Shh* mRNA expression. It is possible that the intermediate SHH protein-based feedback loop continues to function in concert with the slow *Shh*/*Grem1*/*Fgf* loop, since transcriptional regulation of *Shh* may not be sufficient to ensure that correct levels of SHH protein will continue to be produced over the course of limb development. Consistent with this hypothesis, disruption of the SHH protein-based intermediate feedback loop during late limb development results in prolonged *Shh* mRNA transcription (Figure S3 and Bouldin *et al.* 2010) and prolonged variance in the right/left ratio for SHH in limb buds (Figure 3).

Our surprising discovery of SHH protein right/left variance in the early mouse and chick limb buds suggests that variance in right/left SHH protein levels may be evolutionarily necessary. Recently, it has been proposed that patterning of the digits occurs during early limb bud development (during the time we observed SHH protein variance in the limb) (Zhu *et al.* 2008). Our data suggest that digit patterning may not require absolute concentrations of SHH protein to form a given digit. Instead, our observation that normal digit patterning can be obtained in limb buds in which SHH protein levels fluctuate suggests that the presence of a SHH protein gradient may be more important than exposure to a specific concentration of SHH protein for normal digit patterning. It is also possible that the observed fluctuations in SHH protein play a currently unknown role in digit patterning.

## Supplementary Material

Supplemental material is available online at www.g3journal.org/lookup/suppl/doi:10.1534/g3.116.033019/-/DC1.
