# Supplementary figures and images for: SHH Protein Variance in the Limb Bud Is Constrained by Feedback Regulation and Correlates with Altered Digit Patterning
Source: G3 (Bethesda). 2017 Jan 26;7(3):851–8. doi: 10.1534/g3.116.033019 (PMC5345715; doi:10.1534/g3.116.033019)

Fig. S1

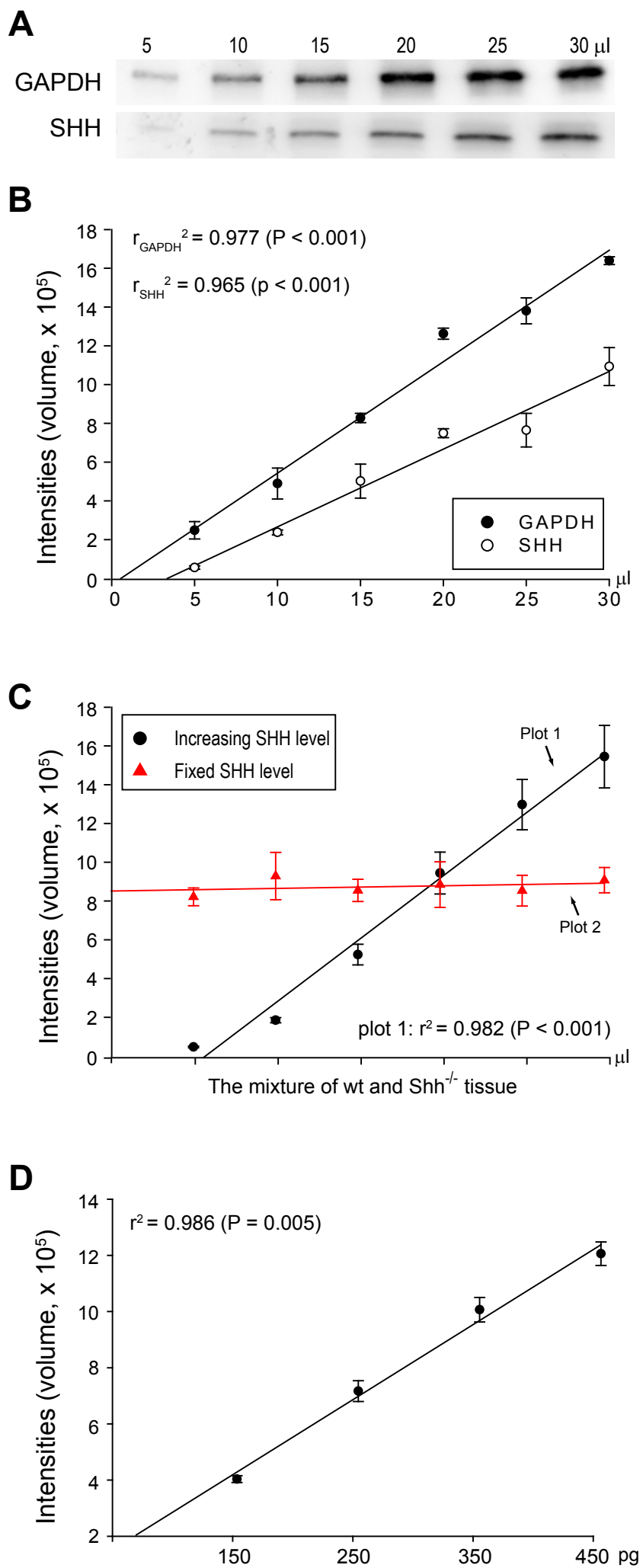

Supplement: Supplementary file 1 [file 851FigureS1.pdf]

Fig. S4

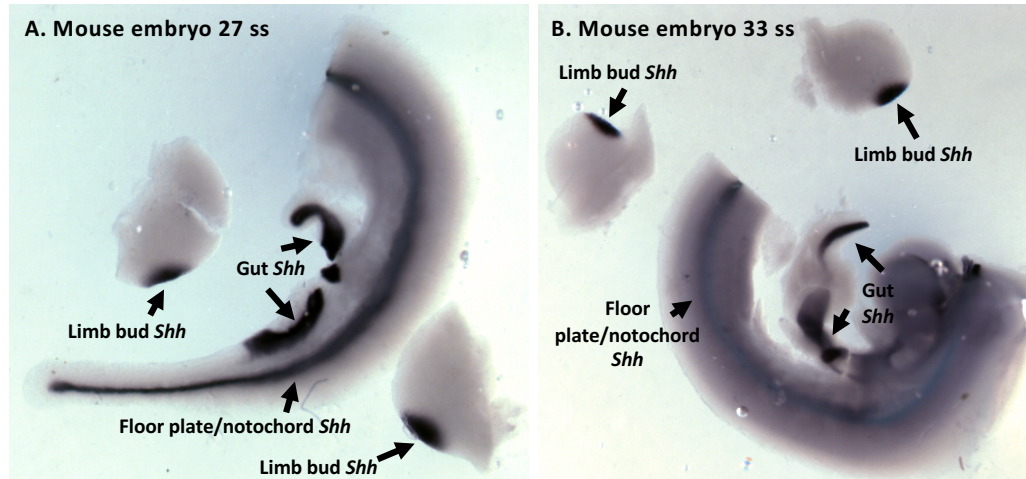

Supplement: Supplementary file 6 [file 851FigureS4.pdf]
