# Supplementary material for: SHH Protein Variance in the Limb Bud Is Constrained by Feedback Regulation and Correlates with Altered Digit Patterning
Source: G3 (Bethesda). 2017 Jan 26;7(3):851–8. doi: 10.1534/g3.116.033019 (PMC5345715; doi:10.1534/g3.116.033019)
Supplement: Supplementary file 5 [file 851FigureS3.docx]

Figure S3. Prolonged *Shh* mRNA transcription in *Msx2-Cre*; *Smo^flox/flox^* limbs. (.ai, 4.72 MB)

<http://www.g3journal.org/lookup/suppl/doi:10.1534/g3.115.020040/-/DC1/FigureS3.ai>
